# Surveillance of serious adverse events following immunization in resource poor settings

**Published:** 2011-01-10

**Authors:** Gabrielle J Breugelmans, Bradford Gessner

**Affiliations:** 1 Agence de Medecine Preventive, c/o Institut Pasteur, 75724 Paris Cedex 15, France

## 

The development of vaccines is one of the most important public health achievements. However, as the incidence of vaccine-preventable diseases has decreased, the general public has become increasingly concerned about vaccine safety. Vaccine safety is evaluated extensively through animal safety studies, clinical trials, and post-licensure surveillance. Safety monitoring in post-licensure surveillance has relied mainly on passive reporting systems such as the Vaccine Adverse Event Reporting System in the United States and epidemiological studies.

Vaccine safety profiles cannot necessarily be generalized to developing countries, where the incidence, type and severity of serious adverse events may differ significantly because of local environmental and genetic influences[1]. With the recent introduction of newly developed vaccines in sub-Saharan Africa (e.g., pneumococcal and rotavirus vaccines), the extensive use of established vaccines in preventive mass vaccination campaigns (e.g., yellow fever vaccine), and the planned introduction of vaccines for use primarily in resource-poor settings (e.g., malaria vaccine), there is an increased need for effective surveillance of serious adverse events following immunization (AEFI). Compared to high-income countries, few low-income economies have functional national pharmacovigilance (PV) systems in place, due primarily to a lack of resources, infrastructure and local PV expertise. The importance of functional national PV systems has recently been underlined with the implementation of yellow fever (YF) mass campaigns as part of the Yellow Fever Initiative (YFI).

The recent and novel use of YF vaccine for preventative mass vaccination campaigns in sub-Saharan Africa has increased the urgency of establishing functional PV systems. Between 2006 and 2013, the YFI will distribute vaccines to residents of high-risk areas in 12 West and Central African countries. A condition of funding from the GAVI Alliance is the implementation of AEFI surveillance. To address this requirement, AMP and WHO have provided technical support, including the development of surveillance tools (e.g., operational guide, notification and investigation forms, standard operating procedures for taking and transporting biological samples), the introduction of active case finding methods, and the creation and training of national expert committees to review and classify suspected serious AEFIs. Reference laboratories, including the Institut Pasteur in Cameroon, and the Robert Koch Institute in Germany provided diagnostic test results to facilitate the YF AEFI classification process. Best practices have been shared between different countries and African-based consultants have been trained to become local experts. Their expertise is now being requested for AEFI surveillance of additional vaccines such as the serogroup A meningococcal conjugate vaccine.
